# Zoledronate-induced acute anterior uveitis: a three-case report and brief review of literature

**DOI:** 10.1007/s11657-021-00964-z

**Published:** 2021-06-28

**Authors:** Xue Jin, Zhangxuan Shou, Yuhong Shao, Pingda Bian

**Affiliations:** 1 Department of Pharmacy, Zhejiang Provincial People’s Hospital, Affiliated People’s Hospital, Hangzhou Medical College, Hangzhou, Zhejiang China; 2 Department of Pharmacy, The Second Affiliated Hospital of Zhejiang Chinese Medical University, Hangzhou, Zhejiang China; 3 Department of Ophthalmology, Zhejiang Provincial People’s Hospital, Affiliated People’s Hospital, Hangzhou Medical College, Hangzhou, Zhejiang China; 4 Department of Geriatrics, Zhejiang Provincial People’s Hospital, Affiliated People’s Hospital, Hangzhou Medical College, No. 158 Shangtang Road, Hangzhou, 310014 Zhejiang China

**Keywords:** Adverse drug reactions, Bisphosphonate, Uveitis, Zoledronate, Zoledronic acid

## Abstract

**Purpose:**

This three-case report aims to highlight the ocular adverse effects induced by bisphosphonate therapy and to call clinicians’ attention.

**Methods:**

Three cases of acute anterior uveitis secondary to the initial dose of zoledronate infusion were reported with focus on their symptoms, treatment regimens, and outcomes. A review of published reports provided a basis for discussion.

**Results:**

Three cases of acute anterior uveitis were either bilateral or unilateral. They demonstrated typical manifestations of bisphosphonate-induced acute anterior uveitis such as eye pain, blurred vision, conjunctival and ciliary hyperemia, keratic precipitates, and flare in the anterior chamber. After topical corticosteroid-containing comprehensive treatments, these symptoms resolved completely without any vision loss and long-term sequelae.

**Conclusions:**

Acute anterior uveitis may be part of the acute phase reaction induced by zoledronate. Patients should be informed of its symptoms in advance and be monitored closely during and after administration. Clinicians should have a good awareness of the zoledronate-associated acute anterior uveitis and to treat it in a prompt and appropriate manner.

## Introduction

Zoledronate, also known as zoledronic acid, is a nitrogen-containing bisphosphonate. Bisphosphonates exert anti-resorptive effect by suppression of osteoclast function via specifically binding to hydroxyapatite at sites of high bone turnover, leading to reduced bone absorption and increased bone mineral density (BMD). Bisphosphonates are indicated for osteoporosis, hypercalcemia of malignancy, Paget’s disease of bone [1], multiple myeloma [2], and metastatic bone cancer from solid tumors [3]. Among bisphosphonates, zoledronate is frequently prescribed for patients who do not tolerate oral bisphosphonates or have metastatic bone disease because of its lower frequency of administration and higher potency. A single dose per year often leads to better compliance for patients who need long-term anti-osteoporosis therapy [4], thereby securing the efficacy of the medication. However, adverse drug reactions were frequently observed following the administration of zoledronate, which included not only the symptoms and signs of an acute phase reaction (APR) such as pyrexia, myalgia, arthralgia, bone pain, and influenza-like illness, but also ocular adverse effects like scleritis, conjunctivitis, and iritis [5, 6]. Despite its rare incidence, acute anterior uveitis (AAU) associated with zoledronate was also documented in literature [7, 8]. AAU is inflammation predominantly of the anterior chamber, iris and anterior vitreous resolving totally within 3 months [9]. In a 70-bed geriatrics ward for older cadres, from November 2011 to December 2020, we prescribed at least one dose of zoledronate for 305 older patients with osteoporosis and observed three cases of AAU. This paper reports these three cases of AAU induced by zoledronate to highlight the ocular adverse effects associated with bisphosphonate therapy and to call clinicians’ attention.

## Case presentation

### Case 1

On December 2, 2014, a 78-year-old woman was given zoledronate (Aclasta®, 5 mg/100 mL solution; Novartis Pharma Schweiz AG, Risch, Switzerland) 5 mg IV for osteoporosis, which lasted for more than 30 min. She had a history of hypertension for more than 10 years and denied any history of bisphosphonate medication or uveitis. In addition to zoledronate, she was routinely given calcium and vitamin D supplements. Twenty-four hours following the completion of infusion, she presented with systematic myalgia, escalated body temperature (up to 38.3 °C), bilateral eye pain, photophobia, and blurred vision. She was advised to drink more water and given levofloxacin eye drops on both eyes. Two days later, her myalgia symptom disappeared and body temperature returned to normal, but the ocular symptoms exacerbated. She was promptly transferred to the Ophthalmology Department of our hospital. Ophthalmological examination showed her visual acuity was 0.5 for the right eye and 0.6 for the left, and the intraocular pressure was 27 mmHg for the right eye and 25 mmHg for the left, respectively. Slit-lamp examination revealed bilateral conjunctival and ciliary hyperemia (+ +), dusty keratic precipitates ( +), flare in the anterior chamber (+ +), and accompanied with fibrin exudation. Fundus examination manifested no anomaly. Laboratory examination revealed serum 25-hydroxyvitamin D (25[OH]D) of 18.2 ng/mL. Then she was diagnosed with bilateral AAU by an ophthalmologist (Yuhong Shao), and started receiving tobramycin/dexamethasone eye drops four times per day, atropine sulfate ophthalmic gel two times per day, and brinzolamide eye drops two times per day. After these treatments, the ocular symptoms gradually resolved. Four weeks later, ophthalmologic examination showed her visual acuity was 1.0 for both eyes, and the intraocular pressure was 13 mmHg for the right eye and 14 mmHg for the left. Slit-lamp examination revealed no conjunctival and ciliary hyperemia, no keratic precipitates, no flare in the anterior chamber, and no fibrin exudation. There was no rechallenge with zoledronate and no recurrence of the symptoms within 6 months of follow-up.

### Case 2

An 87-year-old woman was started on oral alendronate 70 mg weekly along with calcium and vitamin D supplements for osteoporosis. She had a history of chronic gastritis for more than 10 years and denied any history of bisphosphonate medication or uveitis. Three weeks later (January 7, 2019), she was switched to receiving zoledronate (Aclasta®, 5 mg/100 mL solution; Novartis Pharma Schweiz AG, Risch, Switzerland) 5 mg IV, which lasted for at least 30 min. Thirty hours following the completion of administration, she experienced poor appetite, gradually elevated body temperatures (up to 37.8 °C), accompanied with redness, pain, and blurred vision in the right eye. She was then transferred to the Ophthalmology Department. Ophthalmological examination showed her visual acuity was 0.5 for the right eye and 1.0 for the left, and the intraocular pressure was 15 mmHg for the right eye and 16 mmHg for the left. Slit-lamp examination revealed conjunctival and ciliary hyperemia (+ +), dusty keratic precipitates (+ +), flare in the anterior chamber ( +), and fibrin exudation in the right eye. Fundus examination found no anomaly. Laboratory examination revealed serum 25-OHD of 31.7 ng/ml and C-reactive protein of 8.5 mg/dL. Afterwards, a diagnosis of AAU in her right eye was made by the ophthalmologist (Yuhong Shao). She was given tobramycin/dexamethasone eye drops four times per day, and tropicamide/phenylephrine eye drops three times per day immediately. Three weeks after the treatments, all the symptoms in her right eye disappeared. Ophthalmologic examination showed her visual acuity was 1.0 for both eyes, and the intraocular pressure was 12 mmHg for the right eye and 13 mmHg for the left. Slit-lamp examination revealed clear cornea, no conjunctival and ciliary hyperemia, no keratic precipitates, no flare in the anterior chamber, and no fibrin exudation in her right eye. There was no rechallenge with zoledronate and no recurrence of the symptoms within 6 months of follow-up.

### Case 3

On July 21, 2020, a 65-year-old woman was treated with zoledronate (Aclasta®, 5 mg/100 mL solution; Novartis Pharma Schweiz AG, Risch, Switzerland) 5 mg IV for osteoporosis, which lasted for at least 30 min. She had been on alendronate 70 mg weekly along with calcium and vitamin D supplements for 2 years and denied any history of uveitis. Twenty-four hours following the finishing of infusion, she presented with redness, pain, photophobia, and blurred vision in the right eye. She did not experience symptoms like fever and myalgia. She was transferred to the Ophthalmology Department immediately. Ophthalmologic examination showed her visual acuity was 0.8 for the right eye and 1.0 for the left, and the intraocular pressure was 11 mmHg for the right eye and 14 mmHg for the left. Slit-lamp examination revealed conjunctival and ciliary hyperemia (+ +), dusty keratic precipitates ( +), flare in the anterior chamber ( +), no fibrin exudation in the right eye. Fundus examination signified no anomaly. Laboratory examination revealed serum 25-OHD of 13.6 ng/mL. Finally, a diagnosis of AAU in her right eye was made by the ophthalmologist (Yuhong Shao). She then was started on tobramycin/dexamethasone eye drops twice daily. Two weeks later, all the symptoms resolved. Ophthalmologic examination showed her visual acuity was 1.0 for both eyes, and the intraocular pressure was 12 mmHg for the right eye and 13 mmHg for the left. Slit-lamp examination revealed no conjunctival and ciliary hyperemia, no keratic precipitates, no flare in the anterior chamber, and no fibrin exudation in the right eye. There was no rechallenge with zoledronate and no recurrence of the symptoms within 6 months of follow-up.

## Discussion and conclusion

Our report presents three cases of AAU induced by the initial dose of zoledronate infusion. The limitation of this three-case report is that there is no image of the lesions in the involved eyes, making the description of the symptoms and signs of the AAU less instructive.

The Naranjo Adverse Drug Reaction Probability Scale was used to assess the causal relationship between ocular adverse reactions and the potential drug [10]. All the three cases of AUU got a final score of 8, signifying they were very likely to be induced by the infusion of zoledronate, which was featured by (i) AAU was already documented in the specifications for zoledronate injection, and was reported to be connected with the use of zoledronate in the literature [11, 12]; (ii) all the three cases of AAU occurred within 3 days following the administration of zoledronate; (iii) all the three cases demonstrated typical clinical manifestations of AAU such as eye pain, blurred vision, conjunctival and ciliary hyperemia, keratic precipitates, and flare in the anterior chamber; and (iv) there were no other factors could cause the ocular adverse effects alone in all the three cases. The close and clear chronological relationship between zoledronate administration and the onset of ocular symptoms in all our three cases is in line with bisphosphonate-induced ocular inflammations. Furthermore, rapid response to the drug withdrawal and topical corticosteroid application also support a drug-related etiology for this ocular process [2].

All the three cases we report are older women. It may be the result of the predominant number of females with osteoporosis among older people, in combination with the primary indication of zoledronate for the treatment of postmenopausal osteoporosis. According to the findings by Patel DV and colleagues, the mean time from infusion to onset of AAU symptoms was 3 days (range from 2 to 4 days) [13]. Among the three patients, two had taken alendronate tablet before receiving zoledronate infusion and did not experience any AAU symptoms. This tells us long-term oral alendronate tolerance do not exempt patients from ocular adverse effects when receiving intravenous zoledronate [14]. Retrospective cohort studies showed that the yearly incidence for AAU induced by the first dose of oral alendronate was 0.029% [15], while the figure for that induced by the initial zoledronate infusion was 1.1% [13], much higher than the former. In addition, it was also demonstrated that the first-time users of bisphosphonates had an elevated risk of uveitis [15]. Taking into consideration the large population of osteoporosis patients, bisphophonate-related AAU, although rare, should be of great interest to osteoporosis community.

The uveium of the eyes is rich in melanin-related antigens, coupled with abundant and slowly flowing blood in the choroid, making it vulnerable to the invasion of inflammatory mediators. The mechanism underlying zoledronate-induced AAU has not been well-established. Researchers found the trace bisphosphonates secreted into tears might cause ocular inflammation [16]. It was also demonstrated that bisphosphanates could activate γ δ T cells through inhibition of farnesyl pyrophosphate synthase and subsequent intracellular accumulation of isopentenyl diphosphate (IPP) and dimethylallyl diphosphate (DMAPP), thereby resulted in a burst release of proinflammatory mediators such as interleukin-6 and tumor necrosis factor α and led to AAU eventually [17, 18].

Zoledronate-associated AAU can be bilateral or unilateral [19, 20]. Some patients may simultaneously experience the symptoms of the APR such as fever, myalgia, and arthralgia. APR is the most common adverse reaction induced by zoledronate. It usually occurs within the first 3 days following the dosing of zoledronate. All our three cases of AAU were observed within 3 days after the initial administration, suggesting the clinical manifestations of AAU could be a special subset of the signs and symptoms of the APR. This is in accordance with the opinion of some researchers who consider AAU as part of the APR induced by zoledronate [17, 21]. According to Crotti C and colleagues, patients with serum 25(OH)D < 30 ng/mL had a significantly higher risk of APR [22]. In our current report, two patients had 25(OH)D < 30 ng/mL and one had 25(OH)D > 30 ng/mL.

The zoledronate-induced pyrexia, myalgia, arthralgia, and bone pain usually resolve within 4 days of onset, with no need for any interventions. In contrast, the symptoms of AAU will last for a longer time and even deteriorate if they are not treated in a prompt and appropriate manner, leading to further injuries in ophthalmic tissues. Once a patient is diagnosed with zoledronate-induced AAU, he or she routinely needs a corticosteroid-containing therapy, while topical antibiotic may be not effective [7]. Taking our first case as an example, her ocular symptoms deteriorated 2 days after receiving topical levofloxacin alone. According to the finding by Patel and coworkers, the mean duration of topical corticosteroid treatment was 26 ± 10 days (range 17–44) [13], and it should be given on a tapering basis according to the treatment response. If the symptoms are serious at the very beginning or not significantly relieved after topical corticosteroid application, oral or even intravenous corticosteroids should be prescribed [14]. In addition, with the goal to relieve ciliary muscle spasm, reduce exudation, and prevent posterior synechia, cycloplegics such as atropine sulfate eye gel and topicamide eye drops can be applied. As for the patients with increased intraocular pressure, extra medications such as brinzoamine eye drops should be given to lower intraocular pressure. The prognosis for AAU associated with zoledronate is generally good. After careful symptomatic treatment, patients have no risk of vision loss and long-term sequelae.

Orbital inflammation after bisphosphonate administration was also observed. It typically begins 2–6 days after infusion with a concomitant anterior uveitis seen in 29% of reports [4]. Peterson JD et al. reported some degree of bilateral involvement in 29% of cases [23], which might differentiate this entity from idiopathic orbital inflammation. Among bisphosphonates, zoledronate was most reported to be associated with orbital inflammation. Unlike AAU, bisphosphonate-induced orbital inflammation usually need systemic corticosteroid therapy and has the potential for severe and permanent visual compromise [23].

In summary, acute anterior uveitis may be part of the acute phase reaction induced by zoledronate, thereby further complicating zoledronate-associated therapy. Patients should be informed of the symptoms of acute anterior uveitis before receiving zoledronate, enabling them to report to physicians or seek consultation from ophthalmologists immediately after any ocular symptoms present. They also need to be monitored closely during and after the administration of zoledronate. If patients report any ocular symptoms, physicians should take into consideration the possibility of acute anterior uveitis, and refer them to ophthalmologists. Ophthalmologists should also be aware of this ocular adverse reaction given the widespread use of bisphosphonates. Patients presenting with any ocular symptoms should be questioned regarding recent bisphosphonate use. A good awareness of this association by clinicians may allow for an earlier recognition and timely treatment for future cases. Once the diagnosis is established, corticosteroid-containing therapy should be started immediately and withdrawn on a tapering basis according to clinical response. Further clinical studies are warranted to elucidate the possible mechanisms underlying zoledronate-induced acute anterior uveitis and to find effective treatment regimens.

## Method of literature search

A PubMed search was performed with various combinations of the different search terms as follows: zoledronic acid, zoledronate, bisphosphonate, anterior uveitis, and uveitis. Articles in English or with English abstracts were included. Manual search through references from these articles was also done. Additional references of key articles were also included.

## Patient perspective

On follow-up interviews, all the three patients said they really had a great concern about the prognosis for their eyes at the onset of the symptoms, and they were happy about the outcomes after 6 months of follow-up. They thought they received prompt and appropriate treatment, and these events, to some extent, would make them reluctant to receive further bisphosphonate medications, especially the intravenous ones.

## Data Availability

The datasets used and/or analyzed during the current study are available from the corresponding author on reasonable request.
